# Supplementary material for: Prebiotic Peptide Bond Formation Through Amino Acid Phosphorylation. Insights from Quantum Chemical Simulations
Source: Life (Basel). 2019 Sep 16;9(3):75. doi: 10.3390/life9030075 (PMC6789625; doi:10.3390/life9030075)
Supplement: Supplementary file 1 [file life-09-00075-s001.pdf]

Supplementary Material

Prebiotic peptide bond formation through amino acid phosphorylation. Insights from quantum chemical simulations

Berta Martínez-Bachs and Albert Rimola*

Departament de Química, Universitat Autònoma de Barcelona, 08193 Bellaterra, Catalonia, Spain

E-mail corresponding author: albert.rimola@uab.cat

Table S1. Relative potential energies (ΔE), including zero point energy corrections (ΔU_0) and relative free energies at T = 298 K (ΔG_{298}) for the stationary points shown in Figure 3 of the manuscript. Units are in kcal/mol.

Stationary point	ΔE	ΔU_0	ΔG_{298}
AS1-ref	0.0	0.0	0.0
TS-ref	37.2	36.4	48.3
AS2-ref	-5.5	-6.5	-3.5

Table S2. Relative potential energies (ΔE), including zero point energy corrections (ΔU_0) and relative free energies at T = 298 K (ΔG_{298}) for the stationary points shown in Figure 4A and 4B of the manuscript. Units are in kcal/mol.

Figure	Stationary point	ΔE	ΔU_0	ΔG_{298}
4A	AS1-gp	0.0	0.0	0.0
	TS1-gp	42.9	43.4	56.0
	TS2-gp	35.2	35.4	47.8
	AS2-gp	13.7	13.0	11.7
4B	AS3-gp	0.0	0.0	0.0
	TS3-gp	11.1	12.4	24.2
	TS4-gp	8.6	10.1	22.1
	AS4-gp	-9.1	-8.5	-7.5

Table S3. Relative potential energies (ΔE), including zero point energy corrections (ΔU_0) and relative free energies at T = 298 K (ΔG_{298}) for the stationary points shown in Figure 5A and 5B of the manuscript. Units are in kcal/mol.

Figure	Stationary point	ΔE	ΔU_0	ΔG_{298}
5A	AS1-clay	0.0	0.0	0.0
	TS1-clay	31.9	44.4	42.2
	AS2-clay	23.7	22.9	21.8
5B	AS3-clay	0.0	0.0	0.0
	TS2-clay	3.1	4.8	18.6
	I-clay	-4.8	-2.0	13.5
	TS3-clay	6.1	4.8	18.4
	AS4-clay	-5.2	-5.3	-6.1

Table S4. Relative potential energies (ΔE), including zero point energy corrections (ΔU_0) and relative free energies at T = 298 K (ΔG_{298}) for the stationary points shown in Figure 6A and 6B of the manuscript. Units are in kcal/mol.

Figure	Stationary point	ΔE	ΔU_0	ΔG_{298}
6A	AS1-hydr	0.0	0.0	0.0
	R1-hydr	-44.2	-39.5	-22.0
	TS1-hydr	-10.6	-6.9	10.4
	I1-hydr	-66.2	-61.6	-44.5
	TS2-hydr	-45.2	-45.3	-27.9
	P1-hydr	-65.0	-61.2	-42.9
6B	AS2-hydr	0.0	0.0	0.0
	R2-hydr	-34.4	-30.9	-13.8
	TS3-hydr	-22.0	-22.6	-4.7
	I2-hydr	-24.5	-25.1	-7.1
	TS4-hydr	11.2	7.9	26.6
	P2-hydr	-59.3	-54.7	-33.3

Cartesian coordinates of the optimized geometries

Glycin in AS1-ref

6	0.000000	0.555270	0.000000
8	1.168747	0.847656	0.000000
8	-0.995592	1.475724	0.000000
1	-0.585276	2.353769	0.000000
6	-0.560639	-0.861321	0.000000
1	-1.216267	-0.954135	0.871679
1	-1.216267	-0.954135	-0.871679
7	0.421036	-1.925949	0.000000
1	1.024576	-1.857293	0.812594
1	1.024576	-1.857293	-0.812594

TS-ref

7	-0.102072	0.648602	-0.723891
1	-0.253611	0.064525	-1.545607
6	-1.363973	0.960097	-0.050307
6	-2.296067	-0.230909	-0.116038
1	-1.148710	1.205237	0.990590
1	-1.846374	1.821828	-0.517251
8	-2.101092	-1.208126	-0.795373
8	-3.378364	-0.047248	0.655500
1	-3.946527	-0.828691	0.572942
6	0.996049	-0.014419	0.249027
8	0.696858	-0.143420	1.402334
8	1.803145	1.800090	-0.132009
1	0.563369	1.498342	-0.890998
1	2.739961	1.977070	-0.256737
6	1.958354	-0.897326	-0.533335
7	3.197765	-1.175200	0.167331
1	1.428578	-1.842569	-0.713150
1	2.176572	-0.444304	-1.501429
1	2.994961	-1.576068	1.078311
1	3.691381	-0.304219	0.339552

Glycylglycine in AS2-ref

6	2.093787	-0.190125	-0.121610
8	3.026121	-0.862740	-0.475193
8	1.947679	1.086039	-0.497461
1	1.131166	1.474918	-0.104419
6	1.008428	-0.732144	0.827936
1	1.246890	-1.771847	1.038225
1	1.054714	-0.160514	1.761110
7	-0.348077	-0.660196	0.296478
1	-0.840621	-1.497171	0.006641
6	-1.032982	0.492436	0.193100

8	-0.542924	1.596281	0.451355
6	-2.470071	0.374599	-0.305015
1	-3.071915	1.042484	0.320852
1	-2.473288	0.806543	-1.309453
7	-2.948618	-1.008742	-0.359418
1	-3.451391	-1.269281	0.480869
1	-3.560680	-1.167801	-1.149313

Triphosphate (TP) in AS1-gp

15	2.043044	-0.617969	0.036454
8	3.324382	-0.715892	-0.899913
1	4.021206	-0.097905	-0.645767
8	1.382719	-2.014590	-0.168533
1	0.384186	-2.043642	-0.253641
8	1.041189	0.436947	-0.757277
8	2.289161	-0.077288	1.399785
15	-0.015624	1.451920	-0.069757
8	-0.758835	2.246253	-1.059684
8	0.748460	2.194266	1.084082
1	1.370121	1.585954	1.552084
8	-1.014502	0.426672	0.728818
15	-1.998680	-0.661494	0.016015
8	-3.143906	-0.914921	1.083955
1	-3.026453	-1.739494	1.572844
8	-2.745267	0.140276	-1.119895
1	-2.300760	0.964433	-1.409019
8	-1.233325	-1.878748	-0.360986

TS1-gp

15	-3.200366	-0.295507	-0.609527
8	-4.636301	-0.790632	-0.122655
1	-5.294716	-0.085646	-0.154581
8	-2.511800	-1.640147	-1.013869
1	-1.579975	-1.787679	-0.687502
8	-2.450765	0.176407	0.775683
8	-3.220036	0.848444	-1.560708
15	-1.151266	1.169923	0.771306
8	-0.615747	1.334854	2.153410
8	-1.642657	2.498259	0.063921
1	-2.203507	2.284927	-0.716340
8	-0.119814	0.459355	-0.175142
15	0.986812	-1.144451	0.439053
8	2.322242	-2.074111	0.490908
1	2.136859	-2.921907	0.067685
8	1.192917	-0.477154	1.837009
1	0.533721	0.262274	2.104473
8	-0.078064	-2.051834	-0.079688

6	3.306664	0.624302	-0.873776
8	3.371974	1.691624	-1.384235
8	2.015648	-0.026528	-0.887583
1	1.317889	0.633676	-1.126057
6	4.408828	-0.184224	-0.221414
1	4.109002	-0.383993	0.810346
1	4.424998	-1.164989	-0.702988
7	5.710871	0.435275	-0.264073
1	5.729884	1.309104	0.249368
1	6.008311	0.629081	-1.213638

TS2-gp

15	2.528113	-1.017410	-0.735672
8	3.946860	-0.936968	-1.456842
1	4.674551	-1.224409	-0.891306
8	1.533592	-1.018038	-1.939158
1	0.805807	-0.325668	-1.925735
8	2.350758	0.454681	-0.027712
8	2.413955	-2.064410	0.317066
15	1.344854	0.751956	1.222195
8	1.377304	2.185026	1.601907
8	1.761717	-0.289847	2.341570
1	2.022581	-1.152931	1.940202
8	-0.091086	0.305209	0.703840
15	-1.121700	1.384689	-0.697600
8	-2.494237	2.024262	-1.253221
1	-2.959568	1.410416	-1.833878
8	-0.773512	2.670745	0.136523
1	0.033929	2.648323	0.733773
8	-0.338338	0.797624	-1.820480
6	-2.558238	-1.109218	0.594260
8	-2.463825	-0.130283	-0.181654
8	-1.665040	-1.449474	1.457899
1	-0.878530	-0.814700	1.332005
6	-3.799444	-1.990753	0.565305
1	-3.442710	-3.011625	0.393535
1	-4.194521	-1.988731	1.586900
7	-4.842553	-1.664104	-0.372600
1	-4.531594	-1.732758	-1.334211
1	-5.220156	-0.736111	-0.222206

Diphosphate in AS2-gp

15	-1.401552	-0.064004	-0.009716
8	-1.344925	-1.198099	-0.961802
8	-1.590554	1.406157	-0.575411
1	-0.784814	1.724856	-1.023265
8	-2.531687	-0.105771	1.105116

1	-2.873123	-0.996858	1.250494
8	-0.000012	-0.000111	0.843743
15	1.401514	0.063980	-0.009624
8	1.345135	1.198293	-0.961474
8	2.531420	0.105708	1.105453
1	2.876234	0.996066	1.247284
8	1.590332	-1.406081	-0.575704
1	0.784595	-1.724489	-1.023791

GMP in AS2-gp

15	-1.454750	-0.027171	0.116813
8	-1.454332	1.309112	-0.747973
1	-0.698586	1.874309	-0.510462
8	-2.332502	-0.986470	-0.808983
1	-2.934231	-1.530511	-0.286230
8	0.064324	-0.673013	-0.126586
8	-1.817649	0.023847	1.540281
6	1.204646	0.069514	-0.020258
8	1.205526	1.272589	0.071577
6	2.435997	-0.819870	-0.040938
1	2.341069	-1.509259	0.804562
1	2.370733	-1.444671	-0.937269
7	3.709313	-0.137723	0.008645
1	3.825087	0.481778	-0.786006
1	3.785184	0.433587	0.843331

TS3-gp

6	-3.500002	-0.537744	-0.151790
8	-3.634769	0.563674	-0.627029
8	-4.452140	-1.194148	0.522841
1	-5.242511	-0.633376	0.564203
6	-2.234873	-1.361707	-0.261434
1	-1.922770	-1.673103	0.738982
1	-2.442408	-2.259217	-0.850268
7	-1.173002	-0.550161	-0.848972
1	-1.525116	0.057434	-1.586130
1	-0.363432	-1.086226	-1.171482
15	2.706352	-0.199058	-0.009950
8	3.259471	-1.152867	1.191155
1	2.922410	-2.052907	1.119924
8	3.449160	-0.778039	-1.349288
1	4.316019	-0.368088	-1.452817
8	1.225764	-0.598547	-0.260342
8	3.045589	1.216274	0.276158
6	-0.294188	0.414042	0.413126
8	-0.591349	0.078712	1.503483
6	-0.026993	1.787843	-0.181422

1	0.874080	2.161207	0.308752
1	0.214153	1.682872	-1.239177
7	-1.141415	2.696669	0.011085
1	-1.292310	2.892749	0.994121
1	-2.009953	2.343879	-0.378358

TS4-gp

15	2.166129	-0.728811	-0.162555
8	3.503180	0.067569	-0.620740
8	2.713377	-2.028863	0.636991
8	1.351586	-1.059686	-1.369691
1	4.044136	0.344204	0.129080
6	0.110179	1.166568	0.734884
8	-0.360732	1.498472	1.776795
8	1.469850	0.096797	0.960312
6	0.555277	2.089850	-0.400486
1	1.493026	2.529195	-0.051610
1	0.778583	1.514864	-1.298951
7	-0.388893	3.149714	-0.706328
1	-0.685660	3.631605	0.135466
1	-1.212406	2.811532	-1.188846
6	-3.254159	-0.933849	-0.064083
8	-2.966132	-1.994320	0.424555
8	-4.482568	-0.606653	-0.506973
1	-5.073351	-1.358850	-0.346388
6	-2.292161	0.229678	-0.231240
1	-2.400428	0.655441	-1.229093
1	-2.572125	0.996310	0.499320
7	-0.907093	-0.166523	0.012707
1	-0.419066	-0.578970	-0.795665
1	-0.867110	-0.854611	0.765380
1	2.951061	-2.740913	0.030553

Phosphate of AS4-gp

15	-0.077475	-0.062088	-0.108080
8	-1.019919	-0.561484	-1.118574
8	0.995375	-1.148817	0.372176
1	1.666049	-0.809561	0.977611
8	0.895098	1.164489	-0.483875
1	0.434387	1.947620	-0.806921
8	-0.782180	0.507269	1.238309
1	-1.645300	0.101607	1.386211

Triphosphate (TP) with Mg^{2+}-$(H_2O)_4$ in AS1-clay

15	-3.218051	-0.578601	0.211559
8	-4.200083	-0.726307	1.464395
1	-4.880900	-0.042756	1.462564

8	-2.578866	-1.980928	0.120739
1	-1.629523	-2.090868	-0.321607
8	-2.039623	0.443997	0.792725
8	-3.829077	0.075156	-0.981832
15	-1.113496	1.402813	-0.128728
8	-0.249814	2.306626	0.676093
8	-2.105324	2.178347	-1.085246
1	-2.848095	1.589356	-1.366811
8	-0.322756	0.399199	-1.076842
15	0.602401	-0.956415	-0.622609
8	1.908503	-0.800010	-1.436034
8	1.024883	-0.659615	0.841013
8	-0.292307	-2.141917	-0.868882
12	2.856233	0.061205	0.286935
8	3.065046	-0.087484	2.424146
8	4.061072	0.752545	-1.358556
8	2.322703	2.033340	0.523393
1	4.948073	0.453957	-1.584366
1	3.446965	0.347869	-2.003235
1	2.624375	2.708096	-0.093100
1	1.331737	2.181309	0.653639
1	2.139314	-0.391575	2.496476
1	3.129976	0.715162	2.954061
8	3.972726	-1.751418	0.207511
1	3.481025	-2.238923	-0.473253
1	4.022781	-2.315302	0.988096

TS1-clay

15	-3.006180	-0.460694	-0.997560
8	-3.924196	-1.671178	-1.545703
1	-4.710087	-1.776866	-0.996581
8	-2.040272	-0.097342	-2.084802
1	-0.681343	0.499681	-2.039257
8	-2.119906	-1.301438	0.206581
8	-3.802965	0.545495	-0.213514
15	-1.453715	-0.555648	1.441856
8	-0.662319	-1.454996	2.327850
8	-2.633061	0.198473	2.189593
1	-3.281056	0.517633	1.502411
8	-0.573484	0.674539	0.840708
15	0.928677	0.451144	-0.581910
8	2.470043	0.311716	-0.471185
8	0.529659	-1.050064	-0.413547
8	0.241551	0.953265	-1.902115
12	2.311047	-1.766373	0.063878
8	1.159203	-3.578538	-0.331256
8	4.335469	-1.330922	0.590428

8	1.837500	-2.000245	2.039515
1	5.101339	-1.694749	0.133266
1	4.276260	-0.391371	0.339464
1	2.354373	-1.771776	2.816263
1	0.863473	-1.783271	2.224452
1	0.349962	-3.054921	-0.490985
1	0.942065	-4.197708	0.374686
8	3.055351	-2.140802	-1.912157
1	2.981780	-1.288381	-2.365820
1	2.542560	-2.777586	-2.425721
6	0.393853	3.186028	0.405740
8	1.155749	2.246369	-0.032425
8	-0.712517	3.020871	0.963337
1	-0.833455	1.749738	1.010671
6	0.940243	4.601370	0.252459
1	0.112926	5.211912	-0.119021
1	1.142989	4.960611	1.267479
7	2.111648	4.780647	-0.582423
1	1.903689	4.516391	-1.540101
1	2.857681	4.164563	-0.276253

Mg-GMP in AS2-clay

15	-0.306268	1.662590	-0.196324
8	-0.473859	1.759913	1.379465
8	1.167311	1.507786	-0.565600
8	-1.306298	2.401553	-0.983897
12	1.599773	-0.442421	0.014122
8	2.171892	-0.485389	-2.034941
8	1.308303	-0.059247	1.956488
8	1.084385	-2.467690	-0.188467
1	1.018802	-0.652285	2.652456
1	0.224650	1.150585	1.805513
1	0.089986	-2.528790	-0.163125
1	1.444380	-3.197135	0.324254
1	1.852221	0.376724	-2.344827
1	1.870268	-1.161660	-2.649908
8	3.570600	-0.277539	0.753690
1	3.149087	0.005144	1.600213
1	4.200044	0.396514	0.478376
6	-1.674836	-0.875565	-0.130172
8	-1.560180	-2.093499	-0.098011
8	-0.594863	-0.133733	-0.385831
6	-2.987075	-0.149280	0.110730
1	-2.842803	0.464008	1.007898
1	-3.125229	0.573083	-0.698759
7	-4.156616	-0.991052	0.259115
1	-4.018611	-1.674973	0.995441

1	-4.336597	-1.511816	-0.592456

TS2-clay

15	-0.983801	-1.318512	-1.148043
8	-1.765182	-2.422239	-0.313850
8	-1.900468	-0.142779	-1.473246
8	0.023368	-1.882238	-2.093919
12	-1.817905	0.853084	0.425106
8	-1.075051	2.419305	-0.790771
8	-2.855421	-0.642011	1.298460
8	-0.616279	1.628848	1.948145
1	-2.842410	-0.904940	2.220836
1	-2.338113	-1.919631	0.357965
1	0.269564	1.164697	1.891671
1	-0.842889	1.739511	2.874705
1	-1.237595	2.078780	-1.681553
1	-0.112135	2.580677	-0.719672
8	-3.830986	1.541599	0.415252
1	-4.018583	0.635692	0.771942
1	-4.321957	1.650207	-0.405359
6	1.118861	-0.750143	0.699700
8	1.520565	0.160650	1.464084
8	-0.116450	-0.468960	0.091520
6	1.269815	-2.211672	1.138313
1	0.474253	-2.349223	1.878755
1	1.050168	-2.896965	0.318482
7	2.555854	-2.530657	1.735877
1	2.887990	-1.757168	2.302754
1	3.261980	-2.767353	1.049866
6	2.397247	1.775317	-0.531495
8	1.680489	2.736008	-0.355955
8	3.630223	1.668183	-0.020919
1	3.768601	2.395240	0.605239
6	2.073636	0.620267	-1.461454
1	1.056827	0.732893	-1.825931
1	2.755472	0.694656	-2.315090
7	2.179302	-0.696323	-0.826285
1	3.127274	-0.919465	-0.539520
1	1.771521	-1.401604	-1.453678

I-clay

15	-0.691467	-1.867669	0.195896
8	-1.499614	-1.879744	1.565515
8	-1.556255	-2.229023	-0.987953
8	0.688487	-2.434622	0.377227
12	-2.394358	0.802438	-0.444024
8	-2.576643	-0.289280	-2.184350

8	-3.176708	0.123434	1.220403
8	-1.460604	2.529245	-0.808255
1	-3.201254	0.537288	2.085648
1	-2.273678	-1.233026	1.516279
1	-0.488671	2.379863	-0.446176
1	-1.741530	3.438294	-0.684000
1	-2.183435	-1.185985	-1.875746
1	-2.148899	-0.047702	-3.010807
8	-4.467477	1.361388	-0.696999
1	-4.635640	0.852450	0.124759
1	-4.943794	0.925028	-1.412201
6	0.660642	0.612292	0.532235
8	0.708919	1.839939	0.136202
8	-0.471110	-0.141740	-0.061790
6	0.652622	0.404471	2.067899
1	-0.390495	0.575204	2.347534
1	0.874511	-0.630234	2.331771
7	1.503972	1.313677	2.819640
1	1.480737	2.239433	2.403911
1	2.465743	1.003481	2.867637
6	4.184221	0.129386	-0.956170
8	3.847983	-0.131790	-2.082043
8	5.454633	0.326332	-0.565519
1	6.029721	0.231593	-1.340774
6	3.226177	0.321743	0.202876
1	3.602543	-0.182355	1.091769
1	3.150202	1.393667	0.404547
7	1.891066	-0.179745	-0.147618
1	1.728267	-1.205486	0.051659
1	1.739861	-0.043711	-1.150642

TS3-clay

15	-0.542001	-1.694658	0.567789
8	-1.138919	-1.421919	2.017847
8	-1.438794	-2.550971	-0.289141
8	0.941236	-2.074707	0.726068
12	-2.509763	0.511450	-0.483799
8	-2.830267	-1.071422	-1.793426
8	-3.065780	0.272849	1.384270
8	-1.807653	2.178809	-1.332655
1	-3.068792	0.908257	2.103054
1	-1.981156	-0.873289	1.930363
1	-0.782883	2.198878	-1.191763
1	-2.167721	3.060274	-1.448171
1	-2.321214	-1.821683	-1.321298
1	-2.531588	-1.059654	-2.707726
8	-4.635116	0.889057	-0.631420

1	-4.672058	0.611728	0.309417
1	-5.145294	0.248635	-1.139893
6	0.723950	0.900096	0.019168
8	0.602677	1.819619	-0.852541
8	-0.528736	-0.192263	-0.165157
6	0.595251	1.306832	1.505224
1	-0.471263	1.488486	1.655560
1	0.861314	0.481165	2.167128
7	1.334884	2.508208	1.871201
1	1.259882	3.203710	1.135698
1	2.316296	2.329465	2.041330
6	4.279879	-0.207445	-0.697025
8	4.090664	-0.819741	-1.715049
8	5.491088	-0.006213	-0.144654
1	6.154760	-0.428712	-0.711602
6	3.197662	0.479941	0.111413
1	3.370618	0.310607	1.174699
1	3.273245	1.554108	-0.087835
7	1.879427	-0.034607	-0.277066
1	1.562885	-1.141833	0.245819
1	1.866302	-0.164203	-1.290787

Phosphate with Mg^{2+}-$(H_2O)_4$ in AS4-clay

15	1.680967	0.004600	0.010049
8	1.626326	-1.401757	0.790615
8	1.683479	-0.170368	-1.498400
8	3.074997	0.641359	0.510629
12	-1.295792	0.146912	0.074637
8	-0.830869	0.012769	-1.941940
8	-1.210966	-1.593936	0.964966
8	-1.831553	2.044646	0.746781
1	-1.209416	-1.813830	1.898415
1	0.688701	-1.705893	0.853933
1	-0.977109	2.386909	1.056798
1	-2.537721	2.384006	1.303686
1	0.191460	-0.075690	-1.968040
1	-1.097171	0.583255	-2.667115
8	-3.276855	-0.628213	-0.177081
1	-2.846559	-1.409683	0.267689
1	-3.639207	-0.925887	-1.017399
8	0.517119	0.868611	0.529349
1	3.748592	0.559982	-0.173698

$Gly(H_2O)_5$ in AS1-hydr

6	-0.156122	-1.342638	-0.030828
8	-0.056480	-1.394399	-1.286698
8	-1.021580	-1.846733	0.703636

1	1.397996	-0.262206	-1.261639
6	0.953329	-0.510006	0.670055
1	0.513375	0.194654	1.373052
1	1.632448	-1.175807	1.202382
7	1.705604	0.227691	-0.393785
1	1.370706	1.218797	-0.448718
1	2.722353	0.184174	-0.271558
8	0.424432	2.610318	-0.276400
1	0.409214	3.439865	-0.760176
8	-1.963303	0.076740	2.317659
1	-2.696600	-0.150692	2.895253
1	-1.680303	-0.758883	1.873904
8	-2.524557	-0.354803	-1.920812
1	-1.685834	-0.860733	-1.859295
1	-3.202179	-0.980803	-1.647490
8	-1.992158	1.630185	-0.010984
8	4.536178	-0.134543	0.138196
1	5.097784	-0.822862	-0.232866
1	5.074813	0.336380	0.782258
1	-2.256451	0.984442	-0.700408
1	-2.113063	1.175984	0.846111
1	-0.526988	2.305590	-0.186455

R1-hydr

15	1.099512	3.571434	-0.670792
8	1.510161	5.120104	-0.610933
1	2.148131	5.300000	0.089906
8	0.371019	3.294415	-1.942092
1	-0.608624	2.112396	-2.261654
8	-0.038747	3.508682	0.614691
8	2.231048	2.679256	-0.180646
15	-0.378167	2.109387	1.293574
8	-1.513879	2.059761	2.236112
8	1.001697	1.610259	1.957546
1	1.742883	1.889989	1.360565
8	-0.481361	1.105861	0.043401
15	-1.654928	0.614227	-1.023422
8	-1.534554	-0.902413	-1.033689
8	-3.028736	0.965499	-0.474571
8	-1.218499	1.305962	-2.355545
12	-3.162853	-1.175565	0.249163
8	-4.979202	-0.261146	0.823618
8	-2.683417	-3.126537	0.746205
8	-2.309878	-0.616811	2.070373
1	-2.219602	-3.358497	1.556256
1	-2.143572	-3.487065	-0.031528
1	-1.387875	-0.942410	2.193878

1	-2.248566	0.344723	2.258942
1	-4.759283	0.658373	0.591866
1	-5.384945	-0.263826	1.696048
8	-4.116998	-2.134441	-1.412585
1	-3.500569	-2.759366	-1.829304
1	-4.446793	-1.568226	-2.119490
6	1.567246	-3.299968	-0.353886
8	1.010879	-2.165478	-0.430849
8	1.142912	-4.385449	-0.777357
1	2.506049	-2.029078	1.922814
6	2.948458	-3.330318	0.337162
1	3.040985	-4.198408	0.987313
1	3.731366	-3.362798	-0.420709
7	3.173512	-2.084868	1.147625
1	4.156842	-2.026813	1.470839
1	2.996855	-1.245923	0.557655
1	0.465213	-1.483590	1.072637
8	0.387676	-1.253292	2.031602
1	0.707886	-0.339358	2.101863
8	-1.410372	-3.560944	-1.462186
1	-1.105620	-2.630715	-1.500946
1	-0.566521	-4.061208	-1.370250
8	2.682680	0.035453	-0.713620
1	2.501362	1.001661	-0.649231
1	1.855151	-0.434728	-0.921920
8	4.971938	-1.220456	-1.393724
8	5.911656	-1.842117	1.065653
1	5.826043	-1.564145	0.128267
1	6.644297	-1.351278	1.446739
1	4.178592	-0.640957	-1.348604
1	5.271302	-1.231223	-2.306558

TS1-hydr

15	1.206246	2.501460	-1.104227
8	1.149236	3.784526	-2.082192
1	1.294189	4.607341	-1.600194
8	1.123280	1.255052	-1.944867
1	0.034033	-0.292926	-2.380978
8	-0.183562	2.742041	-0.227943
8	2.342276	2.601145	-0.092558
15	-0.474800	2.044767	1.208255
8	-1.841530	2.414816	1.706257
8	0.682098	2.649421	2.153004
1	1.507461	2.721859	1.619519
8	-0.168273	0.520309	1.106836
15	-0.820691	-0.707940	-0.483583
8	-1.807194	-1.694324	0.174320

8	-1.716020	0.520755	-0.816620
8	-0.358562	-1.108376	-2.004823
12	-3.409892	-0.317149	0.036619
8	-4.054281	1.702139	-0.378522
8	-4.729085	-1.877007	0.589972
8	-3.380004	0.357236	1.995223
1	-5.690245	-1.880509	0.579794
1	-4.414523	-2.717055	0.185763
1	-3.090175	-0.255295	2.678647
1	-2.776086	1.163802	2.039217
1	-3.210189	1.960635	-0.789750
1	-4.106332	2.266601	0.404322
8	-3.803239	-1.014190	-1.979496
1	-3.676997	-1.980308	-2.005981
1	-3.077675	-0.643575	-2.502946
6	1.024740	-2.656949	0.022352
8	0.714671	-1.370591	0.133753
8	0.357799	-3.563457	-0.408206
1	2.625647	-1.263642	1.846250
6	2.435356	-2.918957	0.553735
1	2.364434	-3.642223	1.366581
1	3.022666	-3.368411	-0.247837
7	3.131376	-1.700809	1.039543
1	4.113085	-1.924646	1.285311
1	3.170347	-0.969859	0.293231
1	0.876409	-0.139124	2.259920
8	1.547672	-0.474489	2.906773
1	1.747004	0.283988	3.464854
8	-3.223861	-3.571821	-0.888833
1	-2.435514	-3.099093	-0.510273
1	-2.955476	-4.475722	-1.075519
8	3.496303	0.278368	-0.895572
1	3.368274	1.149980	-0.447464
1	2.740195	0.310911	-1.519835
8	6.002625	-0.723725	-1.173497
8	5.850953	-2.314908	1.011039
1	6.070557	-1.800944	0.200956
1	6.599273	-2.226199	1.607440
1	5.133946	-0.266976	-1.233340
1	6.332435	-0.829390	-2.069483

I1-hydr

15	1.449334	1.964105	-1.686450
8	1.119692	2.990059	-2.890497
1	1.338493	3.900405	-2.658288
8	1.310021	0.564691	-2.224898
1	0.197426	-0.480549	-2.633156

8	0.216448	2.302450	-0.636457
8	2.736755	2.321960	-0.963520
15	0.341168	2.431538	1.010197
8	-0.943003	3.110931	1.450636
8	1.576645	3.426991	1.216566
1	2.295708	3.176113	0.590052
8	0.630995	1.053900	1.579829
15	-1.203092	-1.561470	-1.387404
8	-2.112419	-2.747666	-1.493620
8	-1.766555	-0.299768	-0.749213
8	-0.455364	-1.241264	-2.731073
12	-2.999700	0.486411	0.528401
8	-2.709394	2.277176	-0.538800
8	-3.019025	-1.148477	1.836823
8	-2.854184	1.650551	2.194956
1	-2.134808	-1.284155	2.241778
1	-3.334974	-1.978285	1.433909
1	-2.927215	1.328817	3.096659
1	-2.080485	2.304665	2.110828
1	-2.030384	2.053615	-1.195260
1	-2.261777	2.943331	0.021722
8	-4.842186	-0.123204	-0.172228
1	-4.849005	-1.113132	-0.247980
1	-5.240273	0.241872	-0.968567
6	0.993222	-2.870750	-0.444923
8	0.060510	-1.913679	-0.292602
8	1.043576	-3.706937	-1.299892
1	1.054936	-1.472382	1.976276
6	2.041216	-2.780772	0.657665
1	1.923451	-3.637258	1.323589
1	3.031222	-2.835034	0.208634
7	1.945788	-1.533761	1.460875
1	2.750244	-1.485486	2.121576
1	1.997558	-0.701636	0.858892
1	-0.171942	-0.001877	2.392845
8	-0.501711	-0.841308	2.855729
1	-0.406611	-0.683476	3.800528
8	-4.248586	-2.715628	-0.085775
1	-3.441390	-2.807118	-0.697024
1	-4.760312	-3.526574	-0.155074
8	3.496606	-0.288516	-0.676934
1	3.521075	0.692598	-0.619572
1	2.907725	-0.405079	-1.444226
8	5.335520	-1.924138	0.338000
8	4.341577	-1.709798	2.835321
1	4.909842	-1.804518	2.038149
1	4.775887	-1.075394	3.412633

1	4.807538	-1.276667	-0.185995
1	6.158816	-2.083474	-0.130277

TS2-hydr

15	0.350173	2.220357	-1.695740
8	-0.637530	2.796606	-2.842573
1	-0.680724	3.760228	-2.823970
8	0.707255	0.805269	-2.076799
1	-0.117229	-0.520931	-2.004770
8	-0.731575	2.153897	-0.410972
8	1.426147	3.195641	-1.303779
15	-0.342225	2.509835	1.156681
8	-1.624607	2.480796	1.939360
8	0.231163	3.995286	1.058721
1	0.897354	4.033914	0.336403
8	0.808939	1.591167	1.578008
15	-0.662549	-1.896490	-0.363245
8	-1.233149	-3.282127	-0.302846
8	-1.333027	-0.831720	0.506364
8	-0.636869	-1.378205	-1.881572
12	-3.180177	-0.241945	0.446888
8	-3.414975	1.728642	-0.140993
8	-4.542582	-1.699793	0.944853
8	-3.082276	0.404464	2.436668
1	-4.918421	-1.863633	1.813844
1	-4.411308	-2.564125	0.468753
1	-2.619461	-0.223785	3.002972
1	-2.489430	1.216306	2.413174
1	-2.585257	1.967547	-0.603773
1	-3.395245	2.339647	0.612919
8	-3.439163	-0.921558	-1.556525
1	-3.769945	-1.837213	-1.615158
1	-2.590680	-0.939934	-2.040115
6	1.938282	-2.626425	-0.249767
8	0.902783	-1.830040	0.156212
8	1.874317	-3.440035	-1.123826
1	1.960008	-0.832800	2.211081
6	3.187597	-2.349017	0.564671
1	3.305426	-3.193875	1.249825
1	4.031600	-2.372865	-0.129864
7	3.140936	-1.097249	1.337363
1	4.039515	-1.000773	1.817006
1	3.101055	-0.313835	0.678254
1	0.844425	0.596798	2.156632
8	1.118093	-0.453664	2.779262
1	1.449585	-0.219446	3.653409
8	-3.754929	-3.573275	-0.696059

1	-2.747907	-3.583781	-0.568424
1	-4.029862	-4.445930	-0.990553
8	3.274610	0.820126	-1.026152
1	3.158239	1.770731	-0.880058
1	2.464287	0.589205	-1.525120
8	5.605496	-0.571026	-0.992272
8	6.057573	-1.035233	1.688571
1	6.085913	-0.864850	0.725992
1	6.782734	-0.534629	2.071065
1	4.817708	-0.003969	-1.140612
1	6.051365	-0.668522	-1.837170

P1-hydr

15	1.243685	-1.790856	-1.765201
8	2.542225	-0.859740	-2.146019
1	3.250840	-1.430239	-2.471091
8	-0.000778	-1.127872	-2.273538
1	-0.319192	0.459048	-2.599884
8	1.302220	-1.623150	-0.082893
8	1.552793	-3.234350	-2.038424
15	0.903041	-2.749323	1.024620
8	1.595731	-2.399707	2.306071
8	1.289465	-4.138576	0.402376
1	1.410231	-4.076387	-0.592230
8	-0.679050	-2.741266	1.115185
15	-0.102349	2.120001	-1.256721
8	0.447673	3.501860	-1.422744
8	0.625447	1.122611	-0.372273
8	-0.476289	1.441432	-2.633813
12	1.931753	0.867982	1.044231
8	3.336635	-0.416061	1.892982
8	1.891510	2.841848	1.686273
8	0.740514	0.233414	2.607351
1	0.951148	3.092670	1.749787
1	2.291239	3.456210	1.037633
1	-0.235458	0.162121	2.545075
1	1.009716	-0.643764	2.934176
1	4.186699	-0.525435	1.455732
1	2.973484	-1.302646	2.126512
8	3.473054	1.198060	-0.391631
1	3.469941	2.141336	-0.666205
1	3.210222	0.675148	-1.170691
6	-1.842831	2.446482	0.750190
8	-1.647609	2.226356	-0.556197
8	-1.006992	2.847752	1.530350
1	-2.500789	-0.218619	1.842692
6	-3.259377	2.056357	1.129940

1	-3.419882	2.315722	2.175841
1	-3.958113	2.624093	0.505847
7	-3.410920	0.597992	0.949197
1	-4.398106	0.332730	1.032673
1	-3.129620	0.318157	0.008087
1	-1.090440	-1.997449	1.659330
8	-1.784263	-0.759801	2.419563
1	-2.197039	-0.988162	3.259592
8	2.894444	3.860377	-0.665394
1	1.970066	3.830339	-1.061409
1	3.332615	4.650118	-0.994608
8	-2.425994	-1.550485	-1.049664
1	-2.138952	-2.233594	-0.430303
1	-1.630639	-1.386768	-1.598421
8	-5.072917	-1.471920	-1.731967
8	-6.142735	-0.239629	0.468913
1	-5.887083	-0.691730	-0.364379
1	-6.843977	-0.769394	0.857167
1	-4.104320	-1.563694	-1.598663
1	-5.200245	-1.235144	-2.654710

Mg-GMP-hydr in AS2-hydr

15	0.173130	1.278369	0.261464
8	1.564460	1.060947	0.930191
8	0.158775	0.208387	-0.872892
8	-0.241454	2.680426	-0.058385
12	2.058501	-0.492227	-0.431695
8	1.939858	-2.087735	-1.782318
8	4.082124	-0.484300	0.199155
8	1.754721	-1.950702	1.052237
1	4.222653	-1.027200	0.982787
1	4.350700	0.431492	0.418460
1	1.271060	-1.564630	1.806660
1	1.173607	-2.672735	0.735098
1	1.883325	-1.912377	-2.726272
1	1.178554	-2.668660	-1.533250
8	2.740313	0.925563	-1.910303
1	3.258570	1.616781	-1.455417
1	1.931093	1.357654	-2.217080
6	-1.186142	-0.560848	1.729352
8	-1.029298	0.712447	1.319927
8	-0.422694	-1.124370	2.481509
1	-0.888906	-2.758423	-0.394756
6	-2.400106	-1.224611	1.108770
1	-2.663713	-2.102803	1.698159
1	-3.229126	-0.512130	1.094556
7	-2.030048	-1.648535	-0.268723

1	-2.898150	-1.816172	-0.782675
1	-1.554401	-0.874055	-0.742618
8	-0.040225	-3.358818	-0.495793
1	-0.327093	-4.276547	-0.514525
8	3.804677	2.212119	0.332341
1	2.902565	2.005395	0.713857
1	4.097178	3.056040	0.686067
8	-2.805528	3.146866	-0.656311
1	-2.774656	3.552383	-1.526852
1	-1.857393	3.015235	-0.392166
8	-4.549353	1.164660	-0.123378
8	-4.771846	-1.309170	-1.272157
1	-4.778891	-0.391417	-0.925751
1	-5.280760	-1.290617	-2.086432
1	-3.881928	1.861505	-0.343436
1	-5.181687	1.584409	0.465737

R2-hydr

15	1.329888	-0.449457	0.972665
8	2.085465	-1.559382	0.144170
8	2.318871	0.740398	0.937546
8	0.718885	-0.898201	2.262334
12	3.754553	-0.212846	-0.204845
8	4.910006	1.501458	-0.485983
8	4.943036	-1.814934	-0.901354
8	3.173093	0.075397	-2.192268
1	4.835920	-2.017010	-1.837389
1	4.792694	-2.634301	-0.393609
1	2.262019	-0.231654	-2.354317
1	3.145732	1.031412	-2.416157
1	5.372608	1.918361	0.246776
1	4.384016	2.195489	-0.958719
8	4.678459	-0.626148	1.692010
1	4.642643	-1.581403	1.884683
1	4.108400	-0.195177	2.344063
6	0.091049	0.743853	-1.148101
8	0.022881	0.070673	0.046017
8	0.458975	0.204641	-2.161118
1	2.196425	2.934915	-1.418406
6	-0.273188	2.200920	-1.012352
1	-0.542205	2.582672	-1.995998
1	-1.121852	2.285154	-0.333706
7	0.919598	2.928918	-0.497268
1	0.588945	3.827989	-0.136961
1	1.285633	2.433743	0.320197
8	3.097842	2.802813	-1.945208
1	3.184683	3.524324	-2.575363

8	3.857185	-3.230479	1.178753
1	3.017006	-2.766629	0.921137
1	3.628201	-4.031452	1.657942
8	-1.558477	0.122251	3.213278
1	-1.488903	0.185208	4.170869
1	-0.692057	-0.246924	2.900728
8	-2.168163	2.622961	1.949263
8	-0.585107	4.697502	1.202081
1	-1.154609	3.967813	1.540724
1	-0.997990	5.513390	1.494986
1	-1.942314	1.782828	2.387841
1	-2.955877	2.449696	1.400102
6	-3.446180	-0.505578	-1.733991
8	-2.680580	0.391399	-1.276639
8	-4.492051	-0.348598	-2.386440
1	-1.582571	-1.070505	-0.278651
6	-3.022628	-1.960195	-1.425935
1	-3.897258	-2.562306	-1.184239
1	-2.535558	-2.376343	-2.309325
7	-2.062491	-1.980774	-0.287021
1	-2.563129	-2.028310	0.646315
1	-1.336627	-2.706317	-0.378379
8	-3.364700	-1.765416	2.065753
1	-2.831060	-1.132762	2.580705
8	-6.451775	-1.678726	-1.130328
1	-7.332636	-1.626334	-1.510289
1	-5.843956	-1.226121	-1.762355
8	-4.211065	1.997379	0.132512
1	-3.648796	1.503033	-0.525492
1	-4.744946	2.613091	-0.378109
8	-5.521860	-0.358737	1.185182
8	0.320869	-3.429455	-0.841802
1	1.003938	-2.779770	-0.558321
1	0.619397	-4.287913	-0.529759
1	-5.199682	0.492621	0.840609
1	-5.968924	-0.801826	0.438646
1	-4.198671	-1.288030	1.837611

TS3-hydr

15	1.155260	-0.491027	1.205989
8	1.134240	-1.986826	0.921106
8	2.458490	0.198393	0.792704
8	0.706728	-0.082723	2.623750
12	3.972993	-0.209598	-0.361563
8	4.950276	1.613439	-0.358743
8	3.975205	-2.150995	-1.009334
8	3.264859	0.392229	-2.223805

1	3.127190	-2.429443	-1.459867
1	4.067373	-2.806542	-0.296489
1	2.351136	0.093231	-2.386950
1	3.211747	1.370231	-2.288654
1	5.565853	1.986424	0.277057
1	4.351903	2.333409	-0.695732
8	5.036061	-0.883624	1.365316
1	4.619927	-1.742026	1.618826
1	4.826982	-0.294981	2.101179
6	0.089020	0.609394	-1.016261
8	-0.030009	0.283546	0.283614
8	0.597690	-0.117454	-1.845710
1	2.077873	3.013193	-1.160387
6	-0.343874	2.034232	-1.258528
1	-0.334306	2.227779	-2.329926
1	-1.352751	2.160628	-0.867407
7	0.626580	2.929711	-0.570600
1	0.201875	3.859533	-0.493281
1	0.700261	2.626992	0.400447
8	3.053735	3.020005	-1.561543
1	3.157776	3.837128	-2.059266
8	3.477040	-3.102982	1.555649
1	2.542667	-2.776655	1.417231
1	3.433258	-3.877901	2.123271
8	-1.610526	0.505802	2.994943
1	-1.806592	0.579605	3.935103
1	-0.370083	0.185202	2.815163
8	-2.288645	2.879718	1.490873
8	-0.892121	5.020498	0.626084
1	-1.429950	4.298915	1.030077
1	-1.416790	5.821529	0.698018
1	-2.036890	2.107752	2.027545
1	-2.970109	2.572800	0.861425
6	-3.317972	-0.999974	-1.637725
8	-2.506366	-0.061986	-1.374940
8	-4.428477	-0.864869	-2.194112
1	-1.180174	-1.752194	-0.330647
6	-2.893888	-2.419772	-1.227462
1	-3.786358	-2.972092	-0.928182
1	-2.491727	-2.906558	-2.127205
7	-1.933613	-2.394364	-0.111123
1	-2.621990	-1.687280	1.288183
1	-1.517410	-3.307228	0.043547
8	-3.039524	-1.214778	2.078107
1	-2.296296	-0.311584	2.554510
8	-6.400686	-1.818836	-0.684989
1	-7.274117	-1.747516	-1.078718

1	-5.754223	-1.551359	-1.386275
8	-3.986011	1.862539	-0.457372
1	-3.436091	1.168219	-0.937712
1	-4.556084	2.258313	-1.122983
8	-5.264827	-0.095216	1.189548
8	1.513128	-2.868323	-1.645214
1	1.245096	-2.639297	-0.726262
1	0.999106	-2.248661	-2.177996
1	-4.969195	0.638007	0.619023
1	-5.787859	-0.688047	0.611147
1	-3.908428	-0.830096	1.765566

I2-hydr

15	1.188198	-0.442604	1.221991
8	1.112512	-1.947168	1.057316
8	2.497159	0.190712	0.766157
8	0.827422	0.078586	2.655393
12	3.988517	-0.225743	-0.413347
8	4.946118	1.607141	-0.456296
8	3.887052	-2.179630	-1.017501
8	3.229905	0.351195	-2.261370
1	3.003761	-2.460635	-1.396844
1	4.040444	-2.833564	-0.316353
1	2.316336	0.041738	-2.401393
1	3.166312	1.328113	-2.339072
1	5.586619	2.007043	0.136799
1	4.333101	2.314894	-0.797058
8	5.138471	-0.934736	1.227906
1	4.668882	-1.739926	1.557978
1	5.171354	-0.337374	1.983544
6	0.070604	0.604545	-0.987330
8	-0.009679	0.311624	0.332277
8	0.568266	-0.142434	-1.802563
1	2.052945	2.993691	-1.217133
6	-0.367466	2.023705	-1.245650
1	-0.385637	2.194529	-2.320590
1	-1.364493	2.159333	-0.829606
7	0.621154	2.932005	-0.600277
1	0.198282	3.864041	-0.536767
1	0.707643	2.657018	0.377956
8	3.023247	2.985488	-1.639516
1	3.122773	3.791484	-2.156136
8	3.501138	-3.053937	1.623923
1	2.563978	-2.733826	1.517852
1	3.482708	-3.803803	2.225887
8	-1.659738	0.547648	3.080755
1	-1.922253	0.631982	4.003382

1	-0.140505	0.224486	2.859917
8	-2.273965	2.881190	1.497551
8	-0.879476	5.011401	0.618231
1	-1.416582	4.291607	1.027810
1	-1.394759	5.816728	0.707985
1	-2.051686	2.121124	2.064138
1	-2.980055	2.579417	0.892000
6	-3.259270	-0.972277	-1.643985
8	-2.466525	-0.040563	-1.305338
8	-4.344923	-0.813956	-2.240750
1	-1.201813	-1.747095	-0.256240
6	-2.841451	-2.406711	-1.285149
1	-3.743169	-2.978123	-1.058075
1	-2.398241	-2.842261	-2.193359
7	-1.935648	-2.434209	-0.127362
1	-2.711701	-1.760687	1.368552
1	-1.496460	-3.342137	-0.017634
8	-3.113357	-1.291808	2.145920
1	-2.260051	-0.203099	2.684481
8	-6.398196	-1.823216	-0.864174
1	-7.246074	-1.715122	-1.302843
1	-5.711696	-1.530029	-1.513499
8	-4.009216	1.849903	-0.394545
1	-3.439408	1.162866	-0.858134
1	-4.575518	2.224163	-1.075670
8	-5.384600	-0.160973	1.172541
8	1.405921	-2.917164	-1.509141
1	1.155833	-2.678398	-0.589804
1	0.873839	-2.312590	-2.042086
1	-5.047495	0.582168	0.642425
1	-5.856505	-0.738812	0.540821
1	-3.979219	-0.935539	1.829018

TS4-hydr

15	1.227586	-1.123298	0.272671
8	2.281959	-1.265817	-0.908036
8	2.009524	-0.235199	1.293156
8	0.794260	-2.505971	0.780787
12	3.711304	0.006974	0.161087
8	4.526177	1.451872	1.450730
8	5.114307	-0.633625	-1.318487
8	3.411173	1.646867	-1.084700
1	4.745608	-0.369070	-2.180751
1	5.151791	-1.604944	-1.353503
1	2.984714	1.483531	-1.940573
1	3.062983	2.472661	-0.700026
1	4.652804	1.225483	2.376859

1	3.988402	2.280032	1.414120
8	4.449540	-1.592421	1.404797
1	4.471890	-2.410963	0.868795
1	3.686774	-1.692888	1.994533
6	-0.271116	1.754279	-1.034639
8	-0.031883	-0.303566	-0.185056
8	0.607972	2.078802	-1.777299
1	1.820175	3.118792	1.069740
6	-0.615145	2.378012	0.308227
1	-0.823163	3.432008	0.085288
1	-1.523125	1.916677	0.697803
7	0.484811	2.294954	1.284154
1	0.039089	2.372703	2.202021
1	0.876585	1.344010	1.285245
8	2.788176	3.447590	0.869068
1	2.841198	4.390676	1.045812
8	4.086805	-3.185744	-0.791308
1	3.252998	-2.652316	-0.917764
1	3.885946	-4.104808	-0.985340
8	-1.614435	-2.750915	0.961826
1	-1.828899	-3.656380	1.209940
1	-0.525646	-2.668306	0.911774
8	-2.411163	-0.913992	3.089953
8	-1.318815	1.598833	3.467982
1	-1.681205	0.704021	3.273804
1	-1.425343	1.704661	4.416802
1	-2.018583	-1.480948	2.406106
1	-3.351774	-0.883127	2.845710
6	-3.779109	1.275674	-0.747883
8	-3.382248	0.579782	0.219858
8	-4.915002	1.726368	-0.979371
1	-1.125344	0.087797	-0.839391
6	-2.717175	1.600495	-1.817956
1	-3.110594	1.313514	-2.793393
1	-2.552256	2.679145	-1.842940
7	-1.404475	0.906472	-1.606295
1	-2.397349	-1.054707	-1.257047
1	-1.055476	0.530999	-2.486291
8	-2.658256	-1.977132	-1.100966
1	-2.158432	-2.339577	-0.181961
8	-6.545607	-0.234609	-1.896115
1	-7.488699	-0.078576	-1.795928
1	-6.092505	0.580200	-1.580863
8	-4.975386	-0.671320	1.831649
1	-4.456842	-0.050148	1.238338
1	-5.710609	-0.164269	2.188583
8	-5.091326	-2.112817	-0.521396

8	3.008284	-0.126228	-3.143024
1	2.526390	-0.641126	-2.441430
1	2.618426	-0.336291	-3.994525
1	-5.188864	-1.770116	0.393460
1	-5.688051	-1.546748	-1.061919
1	-3.691994	-1.992605	-0.898071

P2-hydr

15	1.104854	-0.840381	-1.496875
8	2.466375	-0.077213	-1.620822
8	0.544334	-0.358723	-0.122704
8	1.331611	-2.366272	-1.503322
12	2.371297	0.201199	0.647365
8	1.920086	0.217534	2.632714
8	4.470208	0.484254	0.688159
8	2.303902	2.276554	0.593569
1	4.677787	1.424984	0.667262
1	4.935014	0.047242	-0.057839
1	2.000756	2.675868	-0.246313
1	1.735831	2.669084	1.287372
1	1.611357	-0.634358	3.025745
1	1.402382	0.970283	2.977430
8	2.911162	-1.974692	0.678504
1	3.831634	-2.103554	0.407921
1	2.381601	-2.361151	-0.068672
6	-1.047974	3.183254	-0.215135
8	0.143887	-0.381297	-2.684320
8	-0.008473	3.718149	0.155636
1	-0.199253	2.810081	2.568981
6	-2.267197	3.218098	0.713480
1	-2.496017	4.275005	0.872362
1	-3.146192	2.735989	0.280605
7	-1.877379	2.624716	2.010547
1	-2.576071	2.842959	2.713725
1	-1.831569	1.604928	1.935060
8	0.737405	2.676585	2.899174
1	0.928909	3.403345	3.498626
8	4.845736	-1.154013	-1.368006
1	3.957693	-0.837680	-1.690112
1	5.363329	-1.424357	-2.130207
8	-0.587501	-3.836478	-0.813904
1	-0.444016	-4.743058	-1.103159
1	0.215000	-3.275845	-1.139238
8	-1.074357	-3.009881	1.924107
8	1.441504	-2.366650	3.160535
1	0.572419	-2.720650	2.894959
1	2.027679	-2.551531	2.410380

1	-0.771531	-3.337600	1.058029
1	-1.166897	-2.034191	1.819662
6	-2.963486	0.791036	-1.542233
8	-2.291795	-0.284057	-1.717161
8	-4.061175	0.864352	-0.965631
1	-0.802418	-0.306370	-2.379859
6	-2.341281	2.089619	-2.093179
1	-2.066051	1.915478	-3.134701
1	-3.107455	2.865660	-2.070629
7	-1.130385	2.531093	-1.404051
1	-2.710518	-1.666362	-1.428432
1	-0.242374	2.478586	-1.901813
8	-2.855331	-2.689385	-1.273065
1	-1.954348	-3.149512	-1.223800
8	-4.459966	-0.532090	1.212885
1	-4.998913	-0.018420	1.820526
1	-4.478755	-0.062007	0.336747
8	-1.528543	-0.335190	1.488955
1	-0.881576	-0.281010	0.738480
1	-2.431282	-0.380973	1.142925
8	-3.756329	-3.146925	1.038617
8	1.808327	2.466420	-2.167568
1	2.136699	1.534160	-2.164453
1	2.263362	2.928650	-2.876171
1	-2.942051	-3.243231	1.574857
1	-4.161489	-2.295694	1.302512
1	-3.295591	-2.873979	-0.347914
